# What do Cochrane systematic reviews say about the use of cannabinoids in clinical practice?

**DOI:** 10.1590/1516-3180.2018.0313210818

**Published:** 2018-10-22

**Authors:** Carolina de Oliveira Cruz Latorraca, Rafael Leite Pacheco, Ana Luiza Cabrera Martimbianco, Daniela Vianna Pachito, Rachel Riera

**Affiliations:** I MSc. Psychologist; Postgraduate Student, Evidence-Based Health Program, Universidade Federal de São Paulo (UNIFESP); and Assistant Researcher, Cochrane Brazil, São Paulo (SP), Brazil.; II MD. Postgraduate Student, Evidence-Based Health Program, Universidade Federal de São Paulo (UNIFESP), and Assistant Researcher, Cochrane Brazil, São Paulo (SP), Brazil.; III MSc, PhD. Physiotherapist; Postdoctoral Student, Evidence-Based Health Program, Universidade Federal de São Paulo (UNIFESP); Professor, Health and Environment Program, Universidade Metropolitana de Santos (UNIMES); Volunteer Researcher, Cochrane Brazil, São Paulo (SP), Brazil.; IV MD, MSc. Neurologist; Postgraduate Student, Evidence-Based Health Program, Universidade Federal de São Paulo (UNIFESP); and Assistant Researcher, Cochrane Brazil, São Paulo (SP), Brazil.; V MD, MSc, PhD. Rheumatologist; Adjunct Professor, Discipline of Evidence-Based Medicine, Escola Paulista de Medicina (EPM), Universidade Federal de São Paulo (UNIFESP); and Researcher, Cochrane Brazil, São Paulo (SP), Brazil.

**Keywords:** Review [publication type], Evidence-based medicine, Evidence-based practice, Cannabis, Cannabinoids

## Abstract

**BACKGROUND:**

The therapeutic effects of cannabinoid compounds have been the center of many investigations. This study provides a synthesis on all Cochrane systematic reviews (SRs) that assessed the use of cannabinoids as a therapeutic approach.

**DESIGN AND SETTING:**

Review of SRs, conducted in the Discipline of Evidence-Based Medicine, Escola Paulista de Medicina (EPM), Universidade Federal de São Paulo (UNIFESP).

**METHODS:**

A broad search was conducted in the Cochrane Database of Systematic Reviews to retrieve any Cochrane SRs that assessed the efficacy and safety of cannabinoids as a therapeutic approach. The results and key characteristics of all reviews included were summarized and discussed.

**RESULTS:**

Eight SRs were included. They assessed the use of cannabinoids for the following types of conditions: neurological (two SRs), psychiatric (two SRs), rheumatological (one SR), infectious (one SR) and oncological (two SRs). There was moderate-quality evidence showing that the use of cannabinoids reduced nausea and vomiting among adults, compared with placebo. Additionally, there was moderate-quality evidence showing that there was no difference between cannabinoids and prochlorperazine regarding the number of participants who reported vomiting, in this same population.

**CONCLUSIONS:**

This review identified eight Cochrane systematic reviews that provided evidence of unknown to moderate quality regarding the use of cannabinoids as a therapeutic intervention. Further studies are still imperative for solid conclusions to be reached regarding practical recommendations.

## INTRODUCTION

More than 500 natural compounds (including cannabinoids, terpenoids and alkaloids) have been identified in the cannabis plant. The recreational and therapeutic effects of cannabinoid compounds (there are nearly 100 of these compounds) have been the center of many investigations. The most common constituent of cannabis is delta-9 tetrahydrocannabinol (THC), the substance that is considered to be the primary psychoactive agent in cannabis.^1^

However, it has been hypothesized that not only THC but also a huge number of other cannabinoids (including synthetic analogues) such as cannabidiol, cannabinol, nabilone, dronabinol and levonantradol have therapeutic effects. The route of administration may play an important role in the effect that cannabis has, and this needs to be considered in designing health interventions. These possible routes involve smoking, vaporization, oral ingestion, passive exposure, intravenous injection and administration of rectal suppositories.^2^

In 2018, a committee designated by the National Academies of Sciences, Engineering and Medicine (NASEM) of the United States described the following as health-related endpoints from medical use of cannabis: therapeutic effects; mental health effects; cannabis abuse; problems relating to cannabis use; cardiometabolic risks; incidence of cancer; and death.^3^

A quick search for cannabis-related trials in the ClinicalTrials database (available at clinicaltrials.gov) in July 2018 showed that 432 studies are currently registered as trials in this database alone. Most of these are investigating the use of cannabis as an intervention for a variety of conditions, such as anxiety, pain, nausea and vomiting, depression and attention-deficit hyperactivity disorder.^4^

Despite the high amounts that have been invested in research on this topic, the relevance of cannabinoids as a therapeutic approach is still a matter of debate. Because these compounds may form a reasonable alternative for treating numerous conditions, it is imperative to assess the efficacy and safety of cannabinoids through well-designed and well-conducted randomized controlled trials.

## OBJECTIVE

To present the evidence from Cochrane systematic reviews that assessed the therapeutic use of cannabinoids for any disease or condition.

## METHODS

### Design

This was a review of Cochrane systematic reviews.

### Setting

This review was conducted within the Discipline of Evidence-Based Medicine of Escola Paulista de Medicina (EPM), Federal University of São Paulo (Universidade Federal de São Paulo, UNIFESP).

### Criteria for including reviews

#### Types of studies

We included the latest version of full Cochrane systematic reviews (SR). We did not consider protocols or any SRs that had the status “withdrawn” in the Cochrane Database of Systematic Reviews (CDSR).

#### Types of participants

We considered participants with any clinical condition, regardless of age or sex.

#### Types of intervention

We considered any intervention derived from cannabis and its synthetic analogues. The cannabinoid compounds considered in these reviews included cannabidiol, cannabinol, nabilone, dronabinol, levonantradol and delta‐9‐tetrahydrocannabinol (THC), in any regimens or doses, when used for therapeutic purposes. We considered any pharmacological or non‐pharmacological intervention as comparators.

#### Types of outcomes

We considered any clinical, social, laboratory or economic outcomes, as assessed and reported in the systematic reviews included.

### Search for reviews

We conducted a broad systematic search in the Cochrane Database of Systematic Reviews (via Wiley) on July 10, 2018. The search strategy is shown in full in Table 1.

**Table 1.** Search strategy in Cochrane Library

#1 MeSH descriptor: [Cannabinoids] explode all trees
#2 MeSH descriptor: [Cannabinol] explode all trees
#3 MeSH descriptor: [Cannabidiol] explode all trees
#4 MeSH descriptor: [Dronabinol] explode all trees
#5 MeSH descriptor: [Cannabis] explode all trees
#6 MeSH descriptor: [Cannabaceae] explode all trees
#7 Cannabinoids or Cannabinol or Cannabidiol or Dronabinol or “9-ene-Tetrahydrocannabinol” or “9 ene Tetrahydrocannabinol” or “delta(1)-Tetrahydrocannabinol” or “delta(9)-Tetrahydrocannabinol” or “Tetrahydrocannabinol” or “Tetrahydrocannabinol, (6a-trans)-Isomer” or “Tetrahydrocannabinol, Trans-Isomer” or “Tetrahydrocannabinol, Trans Isomer” or “Tetrahydrocannabinol, (6aS-cis)-Isomer” or “Tetrahydrocannabinol, Trans-(+-)-Isomer” or “Tetrahydrocannabinol, (6aR-cis)-Isomer” or “THC” or (Cannabis) or Cannabis indica or Cannabis indicas or indicas, Cannabis or Cannabis sativa or Cannabis sativas or sativas, Cannabis or Medicinal Cannabis or Cannabis, Medicinal or Medical Cannabis or Cannabis, Medical or Cannabaceae
#8 #1 OR #2 OR #3 OR #4 OR #5 OR #6 OR #7
Filters: in Cochrane Reviews - Reviews

### Selection of systematic reviews

Two researchers (RLP and COCL) independently read all the abstracts that were retrieved, to check their eligibility in relation to the inclusion criteria. Any disagreements during the selection phase were resolved by a third author (RR).

### Presentation of the results

We produced a synthesis of the key results and characteristics of all the reviews included, using a narrative approach (qualitative synthesis).

For each SR included, we identified the respective population, intervention, comparator and outcomes (PICO); methods for searching for and selecting studies; methods for and results from critical assessment; methods for pooling results (meta-analytic approaches); quality of the body of evidence for each outcome; and applicability. In situations in which multiple interventions were addressed by a single SR, we considered only those that were relevant for the present study.

## RESULTS

### Search results

The initial search retrieved 139 systematic reviews (SRs). After the screening process, 8 SRs were included and brought together in the form of a synthesis of the data.^5^^,^^6^^,^^7^^,^^8^^,^^9^^,^^10^^,^^11^^,^^12^

### Results from systematic reviews

The SRs addressed the following types of conditions: neurological (n = 2),^6^^,^^7^ psychiatric (n = 2),^5^^,^^9^ rheumatological (n = 1),^12^ infectious (n = 1)^8^ and oncological (n = 2).^10^^,^^11^ The main findings from the SRs included and the quality of the evidence (using the GRADE approach) are shown in Table 2. A brief summary of each SR is presented below.

**Table 2:** Characteristics of interventions, comparisons, outcomes and quality of evidence

Population/clinical situation	Number of RCTs	Comparisons	Benefits and harm	Quality of evidence (GRADE approach)*
Dementia^7^	1		Study poorly reported - no conclusion could be drawn	NA
Epilepsy^6^	4	Cannabinoids plus antiepileptic drugs versus antiepileptic drugs alone.	Studies poorly reported - no conclusion could be drawn	NA
Fibromyalgia^12^	2	Dronabinol versus placebo Dronabinol versus amitriptyline.	Benefit of dronabinol • Pain reduction and better quality of life, compared with placebo • Better sleep pattern, compared with amitriptyline	Very low
				Very low
			No difference in: • Pain, mood and quality of life, compared with amitriptyline • Fatigue and depression, compared with placebo	Very low
				Very low
			Harm of dronabinol • Withdrawal due to adverse events • Adverse events	Very low
				Very low
HIV/AIDS patients^8^	7	Dronabinol versus placebo	No difference in: • Likelihood of gaining 2 kg of body weight or more	NA
Nausea and vomiting relating to chemotherapy among adults^11^	23	Cannabinoid versus placebo Cannabinoid versus prochlorperazine	Benefits of cannabinoid over placebo: • Complete absence of vomiting • Complete absence of nausea and vomiting • Withdrawal due to lack of efficacy	Low
				Moderate
				Low
			Harm of cannabinoid • Withdrawal due to adverse event	Very low
			Benefit of cannabinoid over prochlorperazine: • Personal preference: people reported a preference for cannabinoids rather than prochlorperazine	Low
			No difference between cannabinoid and prochlorperazine regarding: • Participants reporting no nausea • Participants reporting no vomiting • Complete absence of nausea and vomiting	Low
				Moderate
				Low
			Harm of cannabinoid: • Withdrawal due to adverse event • Withdrawal due to lack of efficacy • Adverse events	Low
				Very low
				NA
Nausea and vomiting relating to chemotherapy among children^10^	4	Tetrahydrocannabinol versus prochlorperazine/metoclopramide Nabilone versus domperidone	Tetrahydrocannabinol versus prochlorperazine/metoclopramide for reducing nausea: conflicting results among studies included.	NA
			Nabilone versus domperidone: benefit of cannabinoid for reducing nausea.	NA
Schizophrenia^9^	1	Cannabidiol versus amisulpride	No difference between interventions regarding: • Brief Psychiatric Rating Scale-E (BPRS) • Average overall score on Positive and Negative Syndrome Scale for Schizophrenia (PANSS) • Average negative symptom score on PANSS • Average positive symptom score on PANSS	NA
				NA
				NA
				NA
Tourette’s syndrome^5^	2	delta-9-tetrahydrocannabinol (Δ9THC) versus placebo	A positive effect from Δ9THC was reported, but the improvements in tic frequency and severity were small and were only detected through some of the outcome measurements.	NA

RCT = randomized clinical trial; *GRADE (Grading of Recommendations Assessment, Development and Evaluation) aims to assess the quality of the body of evidence. Outcomes are assessed as presenting high quality of evidence (high confidence in results, i.e. the estimated effect is close to the true effect), moderate quality of evidence (it is very likely that the estimated effect is close to the real effect but there is possibility that it is not), low quality of evidence (confidence in the effect estimate is limited) or very low quality of evidence (the true effect is likely to be substantially different from the estimate effect).

#### Dementia

There is some evidence that the cannabinoid system may play a role during the regulation of neurodegenerative processes, including in relation to excessive glutamate production, oxidative stress and neuroinflammation. Neurodegeneration is a feature common to various types of dementia. These findings have led to interest in whether cannabinoids might be useful for treating dementia.

The objective of this review^7^ was to assess the effects of cannabinoids for treating people with dementia. Only one randomized clinical trial (RCT) was included, and the results presented did not provide sufficient data to draw useful conclusions. For further details, refer to the original abstract, available from: http://cochranelibrary-wiley.com/doi/10.1002/14651858.CD007204.pub2/full.

#### Epilepsy

This review^6^ aimed to assess the effects of cannabinoids as monotherapy or add-on treatment for epilepsy and included four RCTs, with 48 participants. Two RCTs were briefly reported as abstract or as letter to the editor. Anti-epileptic drugs were maintained in all studies. The four reports only assessed the secondary outcome of adverse effects. None of the patients in the treatment groups experienced any adverse effects.

Overall, the reports were very poor and precluded any conclusion relating to practice. For further details, refer to the original abstract, available from: http://cochranelibrary-wiley.com/doi/10.1002/14651858.CD009270.pub3/full.

#### Fibromyalgia

Cannabis compounds have been used to reduce pain and other somatic and psychological symptoms. 

This review^12^ assessed the benefits and harm of cannabinoids for treating fibromyalgia symptoms in adults and included two RCTs (72 participants). Both of these studies used nabilone, a synthetic cannabinoid, at a bedtime dosage of 1 mg/day, in comparison with placebo or amitriptyline. Overall, the two studies presented moderate risk of bias. The evidence was derived from grouped mean data on completers (very low-quality evidence overall). The main findings were:

- Pain reduction: greater with nabilone than with placebo; no difference between nabilone and amitriptyline.
- Quality of life: better with nabilone than with placebo; no difference between nabilone and amitriptyline.
- Fatigue and depression: no difference between nabilone and placebo.
- Sleep pattern: greater improvement with nabilone than with amitriptyline.
- Mood: no difference between nabilone and amitriptyline.
- Withdrawal due to adverse events: higher in the nabilone groups (4/52 participants) than in the control groups (1/20 in placebo and 0/32 in amitriptyline group).
- Adverse events: the most frequent adverse events were dizziness, nausea, dry mouth and drowsiness (six participants in the nabilone groups). Neither study reported any serious adverse events.

It was concluded that there was no convincing unbiased high-quality evidence that might suggest that nabilone was useful for treating fibromyalgia. Moreover, its tolerability was low in this population. For further details, refer to the original abstract, available from: http://cochranelibrary-wiley.com/doi/10.1002/14651858.CD011694.pub2/full.

#### HIV/AIDS patients

There have been claims that cannabis improves the appetites of people with AIDS, results in weight gain and lifts mood, thus improving the quality of life. 

This review^8^ assessed the effects of cannabis (in its natural or artificially produced form), either smoked or ingested, on morbidity or mortality among HIV patients. Seven RCTs were included. The evidence that might suggest that cannabis use would have considerable effects regarding morbidity and mortality is currently limited. Data from a single RCT (n = 139, among which only 88 participants were evaluable) that had been conducted at a time before access to highly-active antiretroviral therapy (HAART) became available were assessed. It was found that dronabinol did not provide any benefit regarding the likelihood of gaining 2 kg in body weight or more (RR [risk ratio] 2.09; 95% CI [confidence interval] 0.72 to 6.06).

It was concluded that even though dronabinol has been registered by at least some medicine regulatory authorities for treatment of AIDS-associated anorexia, and even though some jurisdictions make allowances for “medical” use of marijuana by patients with HIV/AIDS, evidence to show that cannabis and cannabinoids would be effective and safe for this purpose is lacking. For further details, refer to the original abstract, available from: http://cochranelibrary-wiley.com/doi/10.1002/14651858.CD005175.pub3/full.

#### Nausea and vomiting relating to chemotherapy among adults

This review^11^ assessed the effects of cannabis-based medications for chemotherapy-induced nausea and vomiting among adults with cancer. In total, 23 RCTs, conducted between 1975 and 1991, were included. No trials involved comparison with newer antiemetic drugs such as ondansetron. The main results from comparisons are summarized below.

Cannabinoid versus placebo:

- Complete absence of vomiting: more frequent with cannabinoid (3 RCTs; 168 participants; RR 5.7; 95% CI 2.6 to 12.6; low-quality evidence);
- Complete absence of nausea and vomiting: more frequent with cannabinoid (3 RCTs; 288 participants; RR 2.9; 95% CI 1.8 to 4.7; moderate-quality evidence);
- Withdrawal due to adverse event: more frequent with cannabinoid (2 RCTs; 276 participants; RR 6.9; 95% CI 1.96 to 24; I^2^ = 0%; very low-quality evidence);
- Withdrawal due to lack of efficacy: more frequent with placebo (1 RCT; 228 participants; RR 0.05; 95% CI 0.0 to 0.89; low-quality evidence).

Cannabinoid versus prochlorperazine

- Participants reporting no nausea: no difference between groups (5 RCTs; 258 participants; RR 1.5; 95% CI 0.67 to 3.2; I^2^ = 63%; low-quality evidence);
- Participants reporting no vomiting: no difference between groups (4 RCTs; 209 participants; RR 1.11; 95% CI 0.86 to 1.44; I^2^ = 0%; moderate-quality evidence);
- Complete absence of nausea and vomiting: no difference between groups (4 RCTs; 414 participants; RR 2.0; 95% CI 0.74 to 5.4; I^2^ = 60%; low-quality evidence);
- Withdrawal due to adverse event: more frequent with cannabinoid (5 RCTs; 664 participants; RR 3.9; 95% CI 1.3 to 12; I^2^ = 17%; low-quality evidence);
- Withdrawal due to lack of efficacy: more frequent with cannabinoid (1 RCT; 42 participants; RR 3.5; 95% CI 1.4 to 8.9; very low-quality evidence);
- Adverse events: dizziness (7 RCTs; 675 participants; RR 2.4; 95% CI 1.8 to 3.1; I^2^ = 12%), dysphoria (3 RCTs; 192 participants; RR 7.2; 95% CI 1.3 to 39; I^2^ = 0%), euphoria (2 RCTs; 280 participants; RR 18; 95% CI 2.4 to 133; I^2^ = 0%), “feeling high” (4 RCTs; 389 participants; RR 6.2; 95% CI 3.5 to 11; I^2^ = 0%) and sedation (8 RCT; 947 participants; RR 1.4; 95% CI 1.2 to 1.8; I^2^ = 31%) were more frequent in the cannabinoid group;
- Personal preference: people reported a preference for cannabinoids rather than prochlorperazine (7 RCTs; 695 participants; RR 3.3; 95% CI 2.2 to 4.8; I^2^ = 51%; low-quality evidence).

Comparisons with metoclopramide, domperidone and chlorpromazine showed weaker evidence, based on fewer trials and participants, for higher incidence of dizziness with cannabinoids. Two RCTs (141 participants) compared an antiemetic drug alone with cannabinoid added to the antiemetic drug and did not show any differences between the groups.

It was concluded that cannabis-based interventions might be useful for adults with refractory chemotherapy-induced nausea and vomiting. However, the methodological limitations of the RCTs reduced the confidence regarding these findings. Future research considering the current chemotherapy regimens and newer antiemetic drugs is likely to modify these conclusions. For further details, refer to the original abstract, available from: http://cochranelibrary-wiley.com/doi/10.1002/14651858.CD009464.pub2/full.

#### Nausea and vomiting relating to chemotherapy among children

This review^10^ assessed the effects of pharmacological interventions for controlling anticipatory, acute and delayed nausea and vomiting among children and young people (aged less than 18 years) who were about to receive or were receiving chemotherapy. In total, 34 RCTs were included, but only four were about cannabinoids. The main comparisons and findings are presented below.

- Tetrahydrocannabinol versus prochlorperazine/metoclopramide: two RCTs showed conflicting results and the heterogeneity of the studies included meant that no data could be pooled.
- Nabilone versus domperidone: cannabinoid showed benefit regarding reduction of nausea (nausea severity score 1.5 compared with 2.5; P = 0.0; scale from 0 [none] to 3 [worst]).

It was concluded that cannabinoids might be effective but that they produced frequent side effects. The current evidence relating to the use of cannabinoids for this purpose is too scarce for any sound conclusion to be reached regarding the implications for clinical practice. For further details, refer to the original abstract, available from: http://cochranelibrary-wiley.com/doi/10.1002/14651858.CD007786.pub3/full.

#### Schizophrenia

This review^9^ assessed the effects of cannabinoids for symptom reduction in people with schizophrenia and included one RCT (39 participants) comparing cannabidiol with amisulpride. The main results are presented below.

- Brief Psychiatric Rating Scale-E (BPRS): no difference between the groups at 7 days (MD [mean difference] -1.50; 95% CI -6.54 to 3.54; 1 RCT; 39 participants), 14 days (MD 1.80; CI -4.61 to 8.21; 1 RCT; 39 participants), 21 days (MD 4.20; CI -4.24 to 12.64; 1 RCT; 34 participants) or 28 days (MD 1.10; CI -8.18 to 10.38; 1 RCT; 35 participants).
- Average overall score (Positive and Negative Syndrome Scale for Schizophrenia [PANSS], total endpoint; higher scores = poor): no difference between the groups at 14 days (MD 0.00; CI -10.10 to 10.10; 1 RCT; 39 participants) or 28 days (MD 0.40; CI -13.42 to 14.22; 1 RCT; 35 participants).
- Average negative symptom score (PANSS; higher scores = poor): no difference between the groups at 14 days (MD 1.20; CI -2.13 to 4.53; 1 RCT; 39 participants) or 28 days (MD 2.70; CI -0.92 to 6.32; 1 RCT; 35 participants).
- Average positive symptom score (PANSS; higher scores = poor): no difference between the groups at 14 days (MD 1.20; CI -1.85 to 4.25; 1 RCT; 39 participants) or 28 days (MD 0.60; CI -3.92 to 5.12; 1 RCT; 35 participants).
- Adverse events: poorly reported and no analyses were performed.

It was concluded that the evidence so far is insufficient to show that cannabidiol has any antipsychotic effect. For further details, refer to the original abstract, available from: http://cochranelibrary-wiley.com/doi/10.1002/14651858.CD004837.pub3/full.

#### Tourette’s syndrome

Gilles de la Tourette syndrome is a developmental neuropsychiatric condition characterized by chronic motor and phonic tics. Currently, the drugs used for Tourette’s syndrome either lack efficacy or are associated with intolerable adverse events.

This review^5^ assessed the effects of cannabinoids for treating tics, premonitory urges and obsessive-compulsive symptoms (OCS), among patients with Tourette’s syndrome. Two RCTs were included (28 participants), and these compared delta-9-tetrahydrocannabinol (Δ9THC), either as monotherapy or as adjuvant therapy, with placebo. One RCT was a double-blind, single-dose crossover trial and the other was a double-blind, parallel-group trial. Both RCTs reported that Δ9THC had a positive effect. The improvements in tic frequency and severity were small and were only detected through some of the outcome measurements.

It was concluded that so far there is not enough evidence to support the use of cannabinoids for treating tics and obsessive-compulsive behavior among people with Tourette’s syndrome. For further details, refer to the original abstract, available from: http://cochranelibrary-wiley.com/doi/10.1002/14651858.CD006565.pub2/full.

## DISCUSSION

This review included eight systematic reviews (SRs) that assessed the use of cannabinoids for neurological, psychiatric, rheumatological, infectious and oncological conditions. Only two of the SRs included assessed the overall quality of the evidence through using the GRADE approach. The only moderate-quality evidence found was related to the use of cannabinoids to treat chemotherapy-related nauseas and vomiting among adults, showing that the use of cannabidiol reduces nausea and vomiting among adults, in comparison with placebo. Additionally, there was moderate-quality evidence showing that there was no difference between cannabinoid and prochlorperazine regarding the number of participants who reported vomiting. All other evidence ranged in quality from low to very low. These findings were similar to those of a previous overview of SRs that addressed only the effects of cannabinoids for nausea and vomiting related to chemotherapy.^13^

The benefits and harm of any therapeutic intervention, including use of cannabinoids, need to be properly addressed through randomized controlled trials (RCTs). Thus, the scope of this review did not extend to presenting results from primary observational or animal experimentation studies. The results from such studies are more susceptible to bias and should always be taken to be exploratory. These studies may nevertheless be useful for guiding well-designed RCTs.

Regarding the implications for practice and research, the results presented in Table 2 may provide guidance for therapeutic proposals. However, it is important to emphasize that, because of the low quality of the evidence, further well-conducted RCTs may change the conclusions regarding the effects of the interventions.

According to these Cochrane SRs, use of cannabinoids to treat medical conditions is not supported by high-quality evidence. The scarcity of data precludes any solid conclusions regarding the efficacy and, especially, the safety of cannabinoids as therapeutic interventions. Further updating of the presented Cochrane systematic reviews also needs to carefully assess the quality of evidence, in order to better support healthcare decisions.

## CONCLUSION

This review identified eight Cochrane systematic reviews (SRs) that provided evidence of unknown to moderate quality regarding the use of cannabinoids as a therapeutic intervention. These SRs found moderate-quality evidence regarding (a) benefits provided by cannabinoids (compared with placebo) for reducing nausea and vomiting that related to chemotherapy among adults and (b) lack of difference between cannabinoids and prochlorperazine regarding the number of participants in this subgroup who reported vomiting.
